# Nationalpopulismus und Faschismus im historischen Vergleich. Zur Aktualität von Max Webers Herrschaftssoziologie

**DOI:** 10.1007/s11609-021-00436-8

**Published:** 2021-05-25

**Authors:** Maurizio Bach

**Affiliations:** Philosophische Fakultät, Universität Passau, Innstraße 41, 94030 Passau, Deutschland

**Keywords:** Herrschaftssoziologie, Faschismus, Populismus, Nationalismus, Charisma, Mittelschichten, Corona-Pandemie, Sociology of domination, Fascism, National socialism, Populism, Nationalism, Charisma, Middle classes, COVID-19 pandemic, Sociologie de la domination, Fascisme, Populisme, Nationalisme, Charisme, Classes moyennes, Pandémie de coronavirus

## Abstract

Der Aufstieg des Rechtspopulismus im vergangenen Jahrzehnt, aber auch jüngst die „autoritär“ anmutenden staatlichen Maßnahmen zur Bekämpfung der Corona-Pandemie haben in verschiedenen politischen Lagern verstärkt Vergleiche mit dem historischen Faschismus evoziert. In dem Beitrag steht die Frage im Mittelpunkt, inwieweit ein solcher diachroner Vergleich empirisch belastbar und damit aussagekräftig ist. Methodisch orientiert sich die Argumentation an Max Webers Konzept des Idealtypus, der als *tertium comparationis* angewendet werden kann. Mit dem „faschistischen Minimum“, einem soziologischen Begriffskonstrukt, das generelle Strukturmerkmale faschistischer Bewegungen und Regime bündelt, steht ein theoretisch tragfähiger und empirisch gehaltvoller Vergleichsmaßstab zur Verfügung. Unter den analytischen Gesichtspunkten des „faschistischen Minimums“ prüft der Artikel, ob und gegebenenfalls in welchem Maße gegenwärtige Tendenzen der Politik und des Staates in Deutschland „faschistische“ Züge aufweisen.

## Abstract (en)

The rise of rightwing populism in the last decade, but more recently also the seemingly “authoritarian” measures taken by the state in protection against the COVID-19 pandemic, inspire ever more frequent comparisons with historical fascism. The paper discusses to what extent such a diachronic comparison is empirically and methodologically sound. The analysis is based in Max Weber’s concept of “ideal type”, which can be used as a *tertium comparationis*. The concept of “fascist minimum”, which systematizes the structural features of fascist movements and regimes, provides a standard of comparison that combines theoretical rigor and empirical substance. Applying the concept of “fascist minimum”, the article examines if and to what extent current tendencies of and in German politics deserve to be called “fascist”.

## Résumé

La montée du populisme de droite au cours de la dernière décennie, mais aussi les récentes mesures étatiques de lutte contre la pandémie de coronavirus perçues comme « autoritaires », ont suscité une augmentation des comparaisons avec le fascisme historique dans différents camps politiques. Cet article pose la question de savoir dans quelle mesure une telle comparaison diachronique est empiriquement solide et par là même pertinente. Sur le plan méthodologique, l’argumentation s’inspire du concept wébérien d’idéal-type qui peut être utilisé comme *tertium comparationis*. Le concept sociologique de « minimum fasciste », qui agrège les caractéristiques structurelles générales des mouvements et régimes fascistes, constitue une échelle de référence théoriquement plus robuste et empiriquement plus consistante. Adoptant la perspective analytique du « minimum fasciste », cet article examine si et, le cas échéant, dans quelle mesure les tendances actuelles de la politique et de l’État en Allemagne présentent des traits « fascistes ».

## Wiederkehr des Bösen?

Erleben wir gegenwärtig mit dem Erstarken der neo-nationalistischen und populistischen Bewegungen in Deutschland und anderen Ländern Europas ein Wiederaufleben des historischen Faschismus und Nationalsozialismus? Kehrt zurück, was wir längst für überwunden gehalten haben: autoritäre Regime mit starken, womöglich charismatischen Führern, die Fremdenfeindlichkeit und Antisemitismus schüren? Befördern völkisch-nationalistische Tendenzen erneut die Selbstzerstörung der Demokratie, wie in den 20er- und 30er-Jahren des letzten Jahrhunderts? Allenthalben wird vor den Gefahren eines neuen Autoritarismus und Faschismus gewarnt, und dabei blicken die Mahner nicht nur auf Ungarn, die Türkei oder Polen. Auch und besonders in Deutschland geraten bestimmte Tendenzen immer wieder unter Faschismusverdacht. In Anbetracht der Wahlerfolge der AfD in den vergangenen Jahren, der Zunahme rechtsradikaler Gewaltakte und der Agitation von neo-nationalsozialistischen Netzwerken im Internet werden hohe Repräsentanten des Staates nicht müde, vor den Gefahren eines neuen Faschismus zu warnen. „Die bösen Geister zeigen sich heute in neuem Gewand“, bemerkte etwa Bundespräsident Frank-Walter Steinmeier in seiner Rede in Yad Vashem im Januar 2020. Und er fuhr fort: „Mehr noch: Sie präsentieren ihr antisemitisches, ihr völkisches, ihr autoritäres Denken als Antwort für die Zukunft, als neue Lösung für die Probleme unserer Zeit. Ich wünschte sagen zu können: Wir Deutsche haben für immer aus der Geschichte gelernt. Aber das kann ich nicht sagen, [...] wenn nur eine schwere Holztür verhindert, dass ein Rechtsterrorist an Jom Kippur in einer Synagoge in Halle ein Blutbad anrichtet. Natürlich: Unsere Zeit ist nicht dieselbe Zeit. Es sind nicht dieselben Worte. Es sind nicht dieselben Täter. Aber es ist dasselbe Böse.“ (Steinmeier 2020)

Es ist freilich nachvollziehbar und erscheint durchaus legitim, wenn angesichts des Holocausts und der Kriegsverbrechen gerade hierzulande immer wieder Vergleiche mit dem Faschismus gezogen werden. Schließlich bleibt der Nationalsozialismus, wie M. Rainer Lepsius einmal bemerkte, ein zentrales „Bezugskriterium für die dauernde Funktionsprüfung von Demokratie, Verfassungsstaat und Sozialordnung“ (Lepsius 2013, S. 174). Bis heute versteht sich die Bundesrepublik in erster Linie als Antwort auf die Perversionen der moralischen Standards, die Auflösung der demokratischen und rechtstaatlichen Ordnung, die Aufhebung der Bürgerrechte und die Gleichschaltung der politischen Öffentlichkeit während der Nazi-Diktatur. Insbesondere durch die Erinnerung an die faschistische Epoche erfolgte somit immer auch eine Bekräftigung der liberalen Wertbeziehungen. Die spätere Abgrenzung gegenüber der zweiten deutschen Diktatur, der DDR, bestätigte die demokratische Antwort im wiedervereinigten Deutschland nur noch einmal.^1^

Dessen ungeachtet sollte aber auch die Frage gestellt werden, ob der immer wieder bemühte Vergleich der gegenwärtigen rechtspopulistischen Tendenzen mit dem historischen Faschismus überhaupt tragfähig, ob er empirisch belastbar ist, und falls ja, in welcher Hinsicht. Es liegt freilich auf der Hand, dass eine diachron-vergleichende Betrachtung nicht direkt und unvermittelt vorgenommen werden kann. Zu offensichtlich sind die Unterschiede zwischen den betreffenden Epochen, etwa hinsichtlich der Gesellschaftsstruktur, des Entwicklungsstandes der Technik, der dominierenden Weltbilder, der Verfassungsordnung und der politischen Realität. Um hier nur wenige Beispiele zu nennen: Die Weimarer Republik war im Vergleich mit der Bundesrepublik eine kaum gefestigte Demokratie, die internationale Arbeitsteilung war generell auf einem weit niedrigeren Niveau als heutzutage, und auch die Massenmedien befanden sich gerade erst am Beginn ihres Aufstiegs, ganz zu schweigen von den heutigen Entwicklungen etwa im Bereich der europäischen Integration oder auch der Digitalisierung, die das damalige Vorstellungsvermögen bei weitem überschritten. Solide historische Vergleiche bedürfen deshalb trennscharfer analytischer Kriterien, die es erlauben, die Grundmuster und -dynamiken unterschiedlicher historischer Phasen möglichst präzise zu erfassen, ohne sich durch deren offensichtliche Differenzen und einzigartigen inneren Verkettungen entmutigen zu lassen.

Dazu bietet sich eine klassische soziologische Methode an: die Bildung von „Idealtypen“ nach Max Weber. Weber zufolge wird ein Idealtypus gewonnen „durch einseitige *Steigerung eines* oder *einiger* Gesichtspunkte und durch Zusammenschluß einer Fülle von diffus und diskret, hier mehr, dort weniger, stellenweise gar nicht, vorhandenen *Einzel*erscheinungen […] zu einem in sich einheitlichen *Gedanken*gebilde“ (Weber 1988, S. 191). Idealtypen sind demnach begriffliche Konstruktionen, die zentrale Struktureigenschaften bestimmter sozialer Erscheinungen oder Konstellationen – „Kapitalismus“, „Bürokratie“, „Monarchie“, „Demokratie“, „Nationalismus“, „Populismus“ u. dgl. – zu empirisch gehaltvollen Kategorien verdichten. Bei den Idealtypen handelt es sich bekanntlich weder um umfassende empirische Beschreibungen der betreffenden Realphänomene noch um reine Fiktionen oder Theorien. Sie dienen in erster Linie als analytische Werkzeuge und damit der Strukturierung des Forschungsproblems. Ihren methodologischen Nutzen erfüllen sie vor allem dann, wenn sie grundlegende Strukturmerkmale des jeweiligen historischen Forschungsobjektes so unterscheiden und kombinieren, als ob sie nur ihrer eigenen Gesetzmäßigkeit und Logik folgen würden, unverfälscht von sonstigen Einwirkungen. Mit anderen Worten, Idealtypen fungieren gleichsam wie konzeptuelle Scherenschnitte, die auf dem Wege der systematischen Gegenüberstellung von tunlichst eindeutig konturiertem Begriff einerseits und konkreter historischer Mannigfaltigkeit andererseits eine analytische Annäherung an die empirische Wirklichkeit ermöglichen.

## Das faschistische Minimum

Die idealtypische Methode hat auch in der soziologischen Faschismusforschung Eingang gefunden und sich dort bewährt. Sie kommt namentlich bei neueren, explizit an Webers Methodologie und Kategorien anknüpfenden Forschungen zum sogenannten „faschistischen Minimum“ zur Anwendung (vgl. Bach und Breuer 2010, S. 17 ff.). Das faschistische Minimum stellt insofern einen Idealtypus im Weberʼschen Sinne dar, als es Gesichtspunkte bündelt, die die Grundlage für einen allgemeinen Begriff des Faschismus abgegeben können. Dabei gelangen sowohl strukturelle Gemeinsamkeiten als auch die inneren Spannungsverhältnisse des historischen Faschismus in den Fokus. Obwohl dem faschistischen Minimum keine historische Wirklichkeit vollkommen entspricht, benennt es doch analytische Minimalkriterien für eine sinnvolle soziologische Verwendung des Faschismusbegriffs als Typuskategorie. In der einschlägigen Literatur finden sich zwei Begriffsvarianten: Einerseits diejenige, die die Bewegungsphase, mithin den Aufstieg der faschistischen Massenparteien im Rahmen der bestehenden konstitutionellen Ordnungen des Königreich Italiens bzw. der Weimarer Republik abbildet. Andererseits diejenige, die auf die sogenannte Regimephase zielt, also auf jene Entwicklungsperiode, die sich an die Übernahme der Regierungsmacht durch die faschistischen Führer anschließt und durch die Etablierung von totalitären Führerdiktaturen bestimmt ist. Da derzeit in Deutschland ein mit der Errichtung faschistischer Führerdiktaturen vergleichbarer Systemwechsel durch rechtspopulistische Bewegungen nicht zu erwarten ist,^2^ sehe ich im Folgenden vom Regimefaschismus ab und betrachte nur die Bewegungsphase, und dies auch nur, um die Gemeinsamkeiten und Differenzen zwischen der heutigen politischen Lage vor allem in Deutschland und der faschistischen Epoche vielleicht etwas klarer zu machen.^3^

Als Bausteine zu einem Idealtypus des Faschismus gelten in erster Linie: a) das öffentliche Bekenntnis zur terroristischen Gewalt und ihre systematische Anwendung, sowohl gegenüber den politischen Gegnern wie gegenüber ausgegrenzten Teilen der Bevölkerung, namentlich den Juden; b) die Herausbildung eines bestimmten Typus politischer Partei, die man im Anschluss an Weber der Kategorie der „Gefolgschaftspartei“ zuordnen könnte; c) die Propagierung eines extremen, völkisch eingefärbten Nationalismus, dessen Massenbasis eine gesellschaftliche Allianz zwischen den deklassierten Mittelschichten und den „Pöbelschichten“ des Volkes (Hannah Arendt) war; d) die (Selbst‑)Zerstörung der parlamentarischen Demokratie durch die ihr eigenen Mechanismen der Führerauslese qua Massenwahlen, der Substitution der politisch verfassten Kollektivität durch das ethnisch definierte („arische“) Volk als Herrschaftsträger; e) ihre Umwandlung in eine totalitäre Führerdiktatur sowie f) eine auf territoriale Eroberung ausgerichtete imperialistische Politik (auf der Grundlage von Bach und Breuer 2010, S. 17 ff. und 413 ff; ergänzt um weitere Gesichtspunkte in Anlehnung an Lepsius 1993b; Arendt 2009, S. 944 ff.). Ubiquitäre Gewaltpraktiken, Gefolgschaftsparteien, plebiszitäre Elitenauslese, extremer Nationalismus, ethnisch-rassische Definition des „Volkes“, charismatische Führerdiktatur sowie Auswüchse eines kontinentalen Imperialismus, der sich in ethnisch-rassischen Panbewegungen manifestiert – so lassen sich die gemeinsamen Grundelemente des Faschismus vor der Übernahme der Staatsmacht zusammenfassend beschreiben. Bezugnahmen auf den historischen Faschismus zur Charakterisierung von destruktiven politischen Gegenwartstendenzen oder zur Warnung vor einem schleichenden oder unverhüllten antiliberalen Zivilisationsbruch sollten sich dieser Elemente des „faschistischen Minimums“ bewusst sein. Jede Evokation des Faschismus als Pervertierung zivilisierter Standards politischer Ordnung, soweit sie sich nicht in wohlfeiler politischer Rhetorik erschöpfen, sondern seriösen wissenschaftlichen Ansprüchen genügen will, sollte die aufgeführten Gesichtspunkte des Idealtypus von Faschismus nicht ignorieren.

Es liegt jedoch auch auf der Hand, dass ein am „faschistischen Minimum“ ausgerichteter Vergleich der aktuellen Tendenzen im Zusammenhang mit dem Rechtspopulismus in Deutschland mit den faschistischen Tendenzen der Zwischenkriegsjahre rasch an seine Grenzen stößt. Heute terrorisieren keine männerbündisch organisierten und bewaffneten paramilitärischen Verbände den öffentlichen Raum, wie seinerzeit die SA oder die *squadre fasciste*, die noch von der Frontgenerationserfahrung des Ersten Weltkrieges geprägt waren und denen vielfach die Rückkehr zu bürgerlichen Lebensformen schwerfiel oder sogar verschlossen blieb. Von vergleichbaren bürgerkriegsähnlichen Zuständen kann heute nirgendwo in Europa die Rede sein. Zwar ist in Deutschland unbestreitbar eine nennenswerte Zunahme von Fällen rechtsradikaler Gewalt festzustellen: Brandanschläge auf Flüchtlingsheime, Verfolgungsjagden auf Migranten in Innenstädten, Enthemmungen fremdenfeindlicher Zusammenrottungen (Freital, Clausnitz), die NSU-Morde, Attentate auf jüdische Einrichtungen (Halle) u.a.m. wären hier zu nennen. Aber die Opferbilanz insgesamt, der Organisationsgrad der Rechten sowie die verfügbaren Waffenarsenale sind mit derjenigen der Bürgerkriegsverhältnisse in den 1920er- und 1930er-Jahren in Italien und Deutschland letztlich nicht vergleichbar. Gegenwärtig handelt es sich, soweit bekannt, ganz überwiegend um radikalisierte Einzeltäter und überschaubare kriminelle Netzwerke, wie die „Reichsbürger“ oder der NSU. Rechte Gewalt kommt im heutigen Deutschland vor allem als ein „Protestverhalten aktiver Mikromilieus“ zum Ausdruck, wie einschlägige Untersuchungen zeigen (vgl. Kohlstruck 2018). Von einer breiteren Mobilisierung von Schlägertrupps und paramilitärischen Organisationen, die die Straße beherrschen, kann derzeit keine Rede sein – was natürlich keinesfalls heißen soll, es gäbe diesbezüglich keinen Grund zur Besorgnis (vgl. Habermas 2020).

Auch Parteien des faschistischen Typs – die zweite analytische Dimension des „faschistischen Minimums“ –, sogenannte personale Gefolgschafts- oder Führerparteien, die illegale Gewaltsamkeit mit friedlicher Stimmenwerbung im Rahmen der Verfassungs- und Rechtsordnung verbinden, sind im rechten Lager im heutigen Deutschland nicht zu erkennen. Die AfD ist ihrem soziologischen Typus nach eine legale und formaldemokratische Programmpartei, die von einem mehrköpfigen Bundesvorstand, den Bundessprechern, geleitet wird. Kein Bundessprecher und keine Bundesprecherin lässt Ambitionen auf eine charismatische Machtergreifung innerhalb der Partei erkennen (auch nicht im sogenannten „Flügel“ um Björn Höcke), die sie in eine absolutistischen Führerpartei verwandeln könnte. Herrschaftssoziologisch würde dies einer Führungsstruktur entsprechen, die „auf der außeralltäglichen Hingabe an die Heiligkeit oder die Heldenkraft oder die Vorbildlichkeit einer Person und der durch sie offenbarten oder geschaffenen Ordnungen“ beruht (Weber 1976, S. 124).

Ob und in welchem Maße rechtspopulistische Politiker in anderen Ländern Europas mit starken nationalistischen Bewegungen, wie etwa Marine Le Pen, Mario Salvini oder Victor Órban, als charismatische Führer im Sinne der Herrschaftssoziologie Webers gelten können, ist eine offene Forschungsfrage. Für die AfD-Führungskader wie Jörg Meuthen, Alexander Gauland, Beatrix von Storch oder Alice Weidel kann das heute aber wohl weitgehend ausgeschlossen werden. Sie repräsentieren eher den traditionellen Typus des parlamentarischen, durch freie Wahlen zu Machtpositionen gelangten Berufspolitikers. Wenn und solange aber das charismatische Element einer nationsweiten politisch-emotionalen Massenmobilisierung fehlt, wäre es irreführend, von einer genuin faschistischen Bewegung oder Partei im engeren Sinne zu sprechen. Denn die Herrschaftsstruktur sowohl des *fascismo* als auch des Nationalsozialismus empfingen ihren Systemcharakter wesentlich durch die politischen Führer an der Spitze, weshalb das Modell der charismatischen Herrschaft als unentbehrlich angesehen wird, um die „spezifische Dynamik faschistischer Regime zu erfassen“ (Bach und Breuer 2010, S. 77; vgl. auch Lepsius 1993b; Bach 1990; Herbst 2010; Wehler 2009).

Hinzu kommt, dass es aktuell in Deutschland offenkundig selbst an einer breiten „latenten charismatischen Situation“ (Lepsius 1993b, S. 100) gebricht. Aus sozialstrukturellen sowie kulturellen Gründen steht eine solche auch in absehbarer Zukunft nicht zu erwarten.^4^ Im deutschen Kaiserreich war schon aufgrund der noch bestehenden größeren Verbindlichkeit von christlichen Traditionen, der Heroisierung von Herrschern wie Friedrich dem Großen oder Otto von Bismarck, des unter den bildungsbürgerlichen Eliten verbreiteten Geniekults sowie der tief im nationalen Habitus verankerten paternalistischen Autoritätsstrukturen die „Bereitschaft, sich im Glauben an ein Charisma einer direkten persönlichen Herrschaft zu unterwerfen“, noch stark ausgeprägt (ebd.). Davon profitierte Hitler in besonderem Maße, der sich ungeniert als neuer Messias inszenierte und die Vorsehung als Narrativ seiner vermeintlichen Unverwundbarkeit evozierte. Heute dagegen dürften die dem Charisma förderlichen kulturellen Dispositionen weitgehend aufgezehrt sein. In einer Kultur des Individualismus und des „Singulären“ (Reckwitz 2017), des Post-Heroismus und Feminismus, wie der unsrigen, wirkt der Ruf nach männlichen Helden als politische Führer doch wie aus der Zeit gefallen (vgl. Müller 2020). Aufgrund des relativ hohen Bildungsniveaus der Bevölkerung sowie vor allem auch der Pluralität an Lebensstilen und Wertvorstellungen kann eine naive persönliche Hingabe- und Opferbereitschaft von großen Teilen der Bevölkerung für nationale, zumal kriegerisch-imperialistische Ziele nicht mehr vorausgesetzt werden. Ein geschlossenes, absolutistisches und im Kern manichäisches Weltbild, das etwa in den Juden die Inkarnation des Bösen und in der arischen Rasse ein „Herrenvolk“ sieht, wie es Hitlers persönlicher politischen Mission zugrunde lag, vermag heutzutage kaum noch sinnstiftend zu wirken. Dies nicht zuletzt deshalb, weil angesichts der vielfältigen und höchst disparaten Wertorientierungen vor allem unter den breiten Mittelschichten, in welchen europäische und kosmopolitische ebenso wie nationale, lokale, religiöse und viele andere Bezüge in mannigfacher und spannungsreicher Schattierung lebendig sind, eine einheitliche und totalitäre Ideologie nur schwerlich verfangen dürfte. Daraus lässt sich schließen, dass die politische Kultur der Gegenwart in Deutschland die Option einer genuin autoritär-charismatischen Führerherrschaft schon aus sozialstrukturellen wie kulturellen Gründen wohl nicht mehr ohne weiteres bereithält.

Aber auch andere Aspekte des „faschistischen Minimums“ wie die expansionistisch-imperialistische Großraumpolitik oder die Umwandlung der konstitutionellen Demokratie in eine monokratische Führerdiktatur faschistischen Typs sind unter den gegenwärtigen gesellschaftlich-politischen Rahmenbedingungen in der Bundesrepublik kaum zu erwarten. Das politische System genießt nach wie vor das Vertrauen der großen Mehrheit der Bevölkerung. Die Zustimmung zu den demokratischen und rechtsstaatlichen Wertbeziehungen ist relativ hoch, wie die allgemeine Wahlbeteiligung und auch repräsentative Meinungsumfragen immer wieder zeigen. Allerdings weist die Systemloyalität bei Teilen der Bürger und Bürgerinnen seit einiger Zeit auch Risse auf. Besonders in der aktuellen Corona-Krise, die die Gesellschaft und die Demokratie vor bisher nicht gekannte Herausforderungen stellt, kommen neuartige Konfliktpotenziale hinzu. Diese resultieren aus der Verknüpfung zahlreicher Faktoren, darunter den einschneidenden Maßnahmen des Staates zur Pandemiebekämpfung, der Bedrohung der wirtschaftlichen Existenz ganzer Branchen und Gewerbe, wie etwa des Einzelhandels und Tourismus, der Überlastung des Gesundheitssystems, den Grenzen der Steuerungsfähigkeit der Bürokratie und den Engpässen bei den Impfkapazitäten. Diese Konflikte könnten das politische System durchaus destabilisieren, auch wenn bisher (Stand April 2021) die Zustimmung der Bevölkerung zu den Anti-Corona-Maßnahmen und damit implizit zu denjenigen Spitzenpolitikern, die einen restriktiven Kurs fordern, ungebrochen relativ hoch zu sein scheint (vgl. Bayerischer Rundfunk 2020; Statista Research Department 2021a).

Der dennoch in Teilen der Bevölkerung bestehende Unmut über die Anti-Corona-Maßnahmen der Regierung artikuliert sich bisher noch weitgehend als diffuser und außerparlamentarischer Protest, ohne feste Parteienstruktur und professionelle Führungskader. So wird die Querdenker-Bewegung, die die Proteste gegen die Maßnahmen wegen der COVID-19-Pandemie in Deutschland wesentlich kanalisiert, in einer von Oliver Nachtwey geleiteten soziologischen Studie als eine höchst widersprüchliche, zum Teil inhaltlich unvereinbare politische Strömungen von Linken über Grüne bis hin zu Reichsbürgern umfassende Bewegung beschrieben, die vor allem durch einen Vertrauensverlust in die Institutionen des politischen Systems, die etablierten Medien sowie die traditionellen Volksparteien gekennzeichnet ist (vgl. Nachtwey et al. 2020; Soldt 2020). In vielbesuchten Internetforen und Videoblogs treten darüber hinaus spontane und zuvor meist unbekannte Aktivisten – darunter viele Ärzte und Anwälte, wie Reiner Füllmich (Rechtsanwalt), Bodo Schiffmann (HNO-Arzt), Raphael Bonelli (Neurologe und Psychiater), Samuel Eckhart, Ulrich Krämer („Doc Ulli“, Internist) –, in Erscheinung, die die einschränkenden Maßnahmen der Regierungen kritisieren. Mit ihrem betont informellen und apolitischen Gestus, einem partizipativen und konsensorientierten Stil sowie ihren ausführlichen, herkömmliche mediale Zeitformate sprengenden^5^ Erläuterungen und Kommentaren zur Entwicklung der Pandemie profilieren sich diese neuen politischen Akteure als elite- und zugleich medienkritische Wortführer des Corona-Protestes. Im virtuellen Raum des Internets gelingt es ihnen, die Zustimmung und Anerkennung einer relativ großen, in die Hundertausende gehenden Zahl von Followern anzusprechen. Diese bilden wohl auch eine nicht unerhebliche Schnittmenge mit den Teilnehmern von Querdenker-Demonstrationen. Es hat den Anschein, als keime in diesen neuartigen Settings einer internetbasierten Gegen- oder Protestöffentlichkeit nicht nur ein neuer Stil politischer Militanz auf. Dieser Stil gibt sich prononciert unpolitisch und spricht überproportional die höheren Bildungsschichten sowie Selbstständige an.^6^ In diesem Zusammenhang treten auch neue – bisher überwiegend männliche – politische Anführer auf, die weder dem politischen Establishment angehören noch bisher öffentlich in Erscheinung getreten sind. Gemeinsam ist diesen Aktivisten die Verknüpfung eines missionarischen Aufklärungsgestus einerseits und einer vorgeblich nüchternen Rhetorik der sachlichen Expertise andererseits. Diese richtet sich in erster Linie gegen den aus ihrer Sicht in der Pandemie von den Regierungen, Gesundheitsagenturen, wie dem RKI, und den öffentlich-rechtlichen Medien an den Tag gelegten quasi apokalyptischen Alarmismus. Möglicherweise handelt es sich hier um eine Form charismatischer Selbststilisierung in embryonaler Gestalt, die auf eine kollektive Zuschreibung einer überlegenen medizinisch-virologischen Expertise, gepaart mit außeralltäglicher Unerschrockenheit und Opferbereitschaft, die auch Züge von Selbstheroisierung trägt, zurückgeführt werden kann.

Doch schon bevor die Corona-Krise akut wurde, zeigte die Systemloyalität von Teilen der bürgerlichen Schichten, besonders im konservativen Lager, Erosionserscheinungen. Diese werden in einer Verschiebung der Wählerpräferenzen hin zu rechtspopulistischen Parteien, wie der AfD, sowie in antidemokratischen und rechtsextremen Einstellungen, die auch in der Mitte der Gesellschaft zunehmend Resonanz finden, offenkundig. Eindeutig in diese Richtung weisen jedenfalls die Ergebnisse der „Mitte-Studie 2018/19“ der Friedrich-Ebert-Stiftung, die in dem Befund zusammengefasst werden können, „dass rechtsextrem-populistische und demokratiefeindliche Einstellungen und Tendenzen in der Mitte [der Gesellschaft, M.B.] tief verwurzelt sind und die Normalisierung rechter Einstellungen sich immer mehr in der Mitte festschreibt und verfestigt“ (Zick et al. 2019, S. 11; siehe auch Decker und Brähler 2020). Dabei handelt es sich freilich nicht bloß um ein Problem der neuen Bundesländer, wo sich in den vergangenen Jahrzehnten eine gewisse Distanz zur bundesrepublikanischen Verfassungsordnung und eine größere Indifferenz gegenüber den Wertbeziehungen der Demokratie verfestigt zu haben scheint, teilweise getragen von kollektiven Ressentiments gegenüber den überwiegend aus dem Westen kommenden Eliten und einem Bewusstsein der individuellen und kollektiven Unterprivilegierung (vgl. Köpping 2018). Auch in Westdeutschland offenbaren robuste Segmente der Mittelschichten eine Affinität zu rechtsradikalen Werten. Darüber hinaus ist festzuhalten, dass selbst Teile der etablierten und gewerkschaftlich organisierten Facharbeiterschaft Neigungen zu einer nationalistischen Radikalisierung zeigen, was freilich nicht nur die Linke irritiert. Die höher qualifizierte Arbeiterschaft zählt sich subjektiv überwiegend selbst zur Mittelschicht, was ihre Neigung erklärt, insbesondere konservative Wertmuster von Letzterer unkritisch zu übernehmen. Eine weitere Säule des Radikalnationalismus bildet das prekäre Dienstleitungsproletariat sowie andere Unterklassen, also das ganze Segment der Unterprivilegierten und Deklassierten (vgl. Dörre et al. 2018; Becker et al. 2018; Reckwitz 2019, S. 239 ff.). Solcherart den radikalen Nationalismus förderliche Allianzen von unterschiedlichen Schichten beschrieb einst Hannah Arendt mit Blick auf den Aufstieg des Totalitarismus als „zeitweiliges Bündnis zwischen Mob und Elite“ (Arendt 2009, S. 702 ff.).

Wenn aktuelle Erscheinungen des politisch-gesellschaftlichen Lebens in Deutschland daher an unheilvolle gesellschaftliche Strukturbedingungen des Aufstiegs faschistischer Bewegungen erinnern, dann sind es wohl vornehmlich die zuletzt genannten ethnisch-nationalistischen Regressionserscheinungen im Zusammenhang mit spezifischen Mentalitätslagen von sozialen Schichten, die dafür zu sprechen scheinen. Dabei tritt wiederum ein wesentlicher Aspekt des oben skizzierten „faschistischen Minimums“ in den Vordergrund: der schichtspezifische Nationalismus des Mittelstandes. Dass die Wahrnehmung von gesamtgesellschaftlichen Krisen einer Radikalisierung des Nationalismus förderlich ist, konnte für die Vergangenheit vielfach belegt werden (Lepsius 1993a; Elias 1989, S. 159 ff.; Wehler 2003, S. 718 ff.). Der historische Musterfall dafür ist wieder vor allem der Aufstieg des Nationalsozialismus. Als besonders anfällig hat sich in der Spätphase der Weimarer Republik, wie übrigens bereits zeitgenössische Beobachter sahen (vgl. u. a. Geiger 1987, S. 109 ff.), gerade der deutsche Mittelstand gezeigt. Schon während des Ersten Weltkrieges bildete er ein „Sammelbecken aller gefährlichen Traditionen und künftigen Giftstoffe“ (Wehler 2003, S. 80), die im gewaltaffinen, ethnozentrisch und rassistisch eingefärbten Nationalismus einen entscheidenden Katalysator fanden. Auch der italienische Faschismus besaß bekanntlich in den mittleren Schichten, den Kleinunternehmern und der staatlichen sowie privaten Angestelltenschaft sein Hauptrekrutierungsfeld (Sylos Labini 1978, S. 73 ff.; Falter 1991, S. 288).

Dass sich gesellschaftliche Widersprüche und Krisen oft in nationalistischen Konflikten entladen, ist freilich keineswegs zufällig. Das lässt sich mit den sozialstrukturellen Eigenarten des Nationalstaates und seinen spezifischen Mechanismen der säkularreligiösen Inszenierung nationalistischer Mythen und Ordnungsvorstellungen für die territorial gebundenen Bevölkerungsmassen erklären. Hierbei kommt gerade den deklassierten Mittelschichten eine entscheidende Rolle zu. Der moderne Nationalstaat hat eine für ihn typische Klassen- und Schichtenstruktur hervorgebracht, die sich von den vormodernen Gesellschaftsverhältnissen grundlegend unterscheidet. An die Stelle relativ starrer geburtsrechtlicher Stände sind dynamische soziale Klassen und „ständische Lagen“ (Max Weber), an die Stelle rechtloser Untertanen des *ancien régime* sind Staatsbürger mit subjektiven Rechten getreten. Der Nationalstaat stellt dabei den allgemeinsten Bezugsrahmen von Gesellschaft für die Bürger dar. Dynastien, Städte und andere lokale Herrschaftsgebilde sind der meist zentralisierten staatlichen Souveränität, die das Gewaltmonopol innehat, untergeordnet. Die aus dem historischen Bürgertum hervorgegangenen Mittelschichten bilden infolgedessen das genuine gesellschaftliche Rückgrat sowie die sozial-moralischen Leitmilieus der nationalstaatsspezifischen Sozialstruktur. Vor allem in ihren konservativen Segmenten weisen sie eine ausgeprägte Affinität zur nationalistischen Weltanschauung und zu den entsprechenden sozialen Praktiken auf. Dies nicht nur, weil sie sich aufgrund ihrer sozialen Mittellage zwischen der Lohnarbeiterschaft und den Eliten in ihren Leistungsnormen und ihren bürgerlichen Wertekanon selbst als Repräsentanten der „Normalmoral“ der Gesellschaft beschreiben, woraus sie einen Anspruch auf Besserstellung und Privilegierung ableiten. Die Empfänglichkeit für nationalistische Empfindungs- und Denkmuster ist vor allem auch darin begründet, dass ihre materiellen Lebenschancen, ihr kulturelles Selbstbewusstsein sowie ihr moralisches Selbstbild in erheblichem Maße von der Macht, der Stabilität und der Prosperität des eigenen Staates respektive der eigenen Nation abhängen. „Das Selbstbewußtsein des Mittelstandes begründet sich“, so M. Rainer Lepsius, „sowohl auf die Definition der eigenen Rolle innerhalb der Nation, wie auf die Definition der Stellung der Nation unter den anderen Nationen. Je größer die Machtentfaltung der Nation nach außen, um so glanzvoller ist dann auch der Geltungsanspruch nach innen“ (Lepsius 1993a, S. 61). Anders ausgedrückt: Geht es dem Staat und der Nation gut, ist es zum Vorteil vor allem der nationalen Mittelschichten – wer immer sonst noch von den günstigen wirtschaftlichen und politischen Umständen profitieren mag.

Der Nationalsozialismus stützte sich aber nicht allein auf den Mittelstand. Es gelang ihm bekanntlich darüber hinaus, auch große Teile das Industrieproletariats für extrem nationalistische Positionen zu gewinnen, und dies, obwohl in der Weimarer Zeit auf Seiten der Linken die internationalistisch-marxistische Ideologie erneut an Deutungsmacht gewonnen hatte, nachdem sie im chauvinistischen Massenenthusiasmus der Kriegsjahre selbst innerhalb der Arbeiterschaft weitgehend untergegangen war. Dabei machten sich die Nationalsozialisten nicht nur den verletzten Nationalstolz infolge der Kriegsniederlage zunutze (Elias 1989, S. 209 ff.). Die Vorspiegelung von erweiterten gesellschaftlichen Teilhabechancen und die Propagierung des Mythos der egalitären Volksgemeinschaft trugen das ihre zur erfolgreichen Nationalisierung der Arbeiterschaft bei. Das spiegelte sich nicht zuletzt in „einem atemberaubenden Ausmaß ihrer Führerloyalität“ wider (Wehler 2003, S. 732). Die klassen- und schichtenübergreifende Deutungskraft nationalistischer Überzeugungen, zu denen eine kollektive Selbstüberhöhung gehört, wirkte allerdings als Erbe des Nationalsozialismus in der Bunderepublik fort. Doch auch in der DDR blieb sie Bestandteil des kollektiven Bewusstseins, trotz des teilungsbedingten und offiziellen Post-Nationalismus in Westdeutschland und des eigenen sozialistischen Internationalismus, den die DDR-Führung zur Staatsideologie erhoben hatte. Im Westen wirkte das relativ hohe Wohlstandsniveau als Kompensation für den amputierten Nationalismus; in der DDR wurde ihm vom Regime ein internationalistisch-marxistisches Mäntelchen umgehängt. Mit der Deutschen Einheit 1990 keimte der Nationalismus schließlich auch in seiner ethnisch-chauvinistischen Färbung rasch wieder auf, und zwar in Form der Wiedervereinigungseuphorie, im Begeisterungstaumel der Fußballweltmeisterschaft von 2006 sowie in der rechtsradikalen Mobilisierung von Fremdenfeindlichkeit und Elitenhass in Ostdeutschland. Schließlich fand er in der „Alternative für Deutschland“ (AfD) im Gefolge der Finanz- und Staatsschuldenkrise am Anfang der 2010er-Jahre eine eigenständige politische Organisationsform, eine neue Sprache und eine eigene parlamentarische Repräsentation, die dem deutschen Rechtsradikalismus ein moderneres, man möchte sagen: „bürgerlicheres“ Profil verliehen. Die vorläufig letzte Stufe der nationalistischen Radikalisierung erfolgte bemerkenswerterweise gleichzeitig mit den Anzeichen einer Schwächung der Europäischen Union im Zusammenhang mit der schwersten Vertrauenskrise des bisher wirkmächtigsten Projekts einer supra-nationalen Neuordnung Europas seit ihren Gründungsjahren. In ihren Anfängen repräsentierte die AfD bekanntlich vor allem jene Angehörigen der Mittelschichten, die sich als vermeintliche Verlierer der von der EU eingeschlagenen Euro-Rettungspolitik sahen und eine Wiederherstellung der nationalen Währungssouveränität forderten (vgl. Schmidt 2016, S. 103 f.; Hirschmann 2017, S. 16 ff.).

## Radikalisierung der deutschen Mittelschichten?

Nun stellt sich aber die Frage, ob man heute noch von einem schichtspezifischen Nationalismus sprechen kann oder ob das nicht etwas kurzschlüssig ist? Besteht tatsächlich nach wie vor eine akute Gefahr der national-chauvinistischen Radikalisierung der deutschen Mittelschichten, eine solche, die über das derzeitige Mobilisierungspotenzial der AfD nennenswert hinausgeht? Ähnlich wie bei der Charisma-Frage sind auch hier Differenzierungen angebracht. Dabei müssen vor allem bestimmte Strukturbesonderheiten spätmoderner Postindustriegesellschaften Berücksichtigung finden. Mit Blick auf die gesamtdeutsche Lage der Mittelschichten stellt sich die Situation im gegenwärtigen Deutschland ebenfalls weitaus uneinheitlicher und komplexer dar als zur Zeit der Weimarer Republik.

Mit dem sich seit den 1980er-Jahren vollziehenden Strukturwandel hin zur Etablierung einer postindustriellen Dienstleitungs- und Digitalökonomie erfahren gerade die Mittelschichten einen weiteren tiefgreifenden Veränderungsprozess. Dieser geht, wie etwa Andreas Reckwitz (2017, 2019) argumentiert, mit einem massiven Schub zur Pluralisierung der Lebensstile und zur Formung eines neuen, spätmodernen Selbst einher, „das sich grundlegend von jenem Sozialcharakter unterscheidet, der die klassische Moderne der Industriegesellschaft dominiert“ (Reckwitz 2017, S. 273). Ohne hier auf den komplexen soziologischen Diskurs über Sozialstruktur und Lebensstile näher eingehen zu können (vgl. schon Müller 1992), sei mit Blick auf den Lebensstil der neuen Mittelklasse nur die zusammenfassende Beschreibung von Reckwitz (2019, S. 92) wiedergegeben: Er „lässt sich auf die (durchaus spannungsreiche) Formel des Strebens nach ‚erfolgreicher Selbstentfaltung‘ bringen. Die zentrale Lebensmaxime lautet hier, individuelle Wünsche und Begabungen zu entfalten, ein Leben zu führen, das man als befriedigend, sinnvoll und reichhaltig empfindet. Zugleich soll es sich um ein erfolgreiches Leben handeln, das mit hohem sozialen Status und sozialer Anerkennung einhergeht.“

Daraus kann in der Gesamtschau gefolgert werden, dass besonders mit Blick auf die Interessen und Werthaltungen die Mittelschichten heutzutage keinen homogenen sozialmoralischen Block mehr bilden. Angesichts des unvergleichlichen Wohlstandes, der in den Nachkriegsdezennien erreicht wurde, versteht sich dies hinsichtlich der materiellen Lebenslage fast von selbst. Es trifft aber vor allem auch mit Blick auf die erwähnten milieuspezifischen Wertorientierungen und Lebensstile zu (vgl. statt vieler Müller 2013). Eine der in dieser Hinsicht virulentesten Bruchlinien geht heute quer durch das breite Segment der Mittelschichten: Sie bildet vor allem die lebensstilbezogene und ideologische Konfrontation zwischen den akademisch qualifizierten urbanen Eliten einerseits und den konservativen Segmenten des Mittelstandes andererseits ab. Erstere vertreten überwiegend einen progressiv-kosmopolitischen und diversitätsaffinen Liberalismus. Sie verkörpern diese politisch-ideologische Haltung auch in ihrem sozialen Habitus. Für Letztere ist dagegen eine Wertschätzung von nationalen Symbolen und Bezügen bzw. eine Rückbesinnung auf sie nach wie vor von sinnstiftender Bedeutung (vgl. Koppetsch 2019, S. 70 ff.). Während für die urban-akademischen Eliten die globale bzw. die europäische Gesellschaft bereits ganz selbstverständlich den relevanten Bezugsrahmen (real oder erwünscht) für die nationale und internationale Krisen- und Konfliktbewältigung darstellt, was nicht zuletzt in ihrer Präferenz für eine vertiefte europäische Integration, offene Grenzen und eine beherzte planetarische Klimapolitik zum Ausdruck kommt, denken konservative Kreise noch vornehmlich in Kategorien territorialer Geschlossenheit und ethnisch-kultureller Homogenität. Die stärker traditionsgebundenen Milieus sind dementsprechend gegenüber der Europäischen Union wenig aufgeschlossen bis ablehnend eingestellt. Deren Engagement für ein integriertes Europa erweist sich auch rückblickend oft nur als Lippenbekenntnis, wie Forschungen über die „lange (vergessene) Geschichte der Euroskepsis“ zeigen (Patel 2020). Sie fordern verschärfte Grenzkontrollen und erwarten vom Staat eine entschiedenere Politik im nationalen Interesse („nation first“), nach innen wie nach außen. Das mobilere, fremden Kulturen gegenüber aufgeschlossenere und eher von den jüngeren Generationen geprägte Segment der Mittelschichten dagegen hat jedoch auch objektiv durch die Globalisierungs- und Europäisierungsprozesse einen Zuwachs an Status‑, Macht- und Wohlstandschancen erfahren. Eine Folge davon ist die Aufwertung eines kosmopolitisch getönten Elitehabitus bei spiegelbildlicher Abwertung nationalistischer Einstellungen. Darüber hinaus lässt sich in diesem Sozialmilieu auch ein deutlich geschärfteres Gewissen und eine erhöhte Sensibilität gegenüber globalen sozialen Ungleichheiten und Risiken beobachten. Die Menschenrechte, der Klimawandel, der Feminismus sowie post-kolonialistische Einstellungen prägen dementsprechend deren politisches Selbstverständnis. Der Großteil der weniger mobilen, konservativen und national orientierten Mittelschichten sieht sich dagegen nicht nur mit einer Entwertung des national tradierten Kultur- und Bildungskapitals konfrontiert (vgl. Kraemer 2018; Schimank et al. 2014). Diese Gruppierungen erfahren darüber hinaus auch eine Herabsetzung bzw. Relativierung ihrer zentralen Wertmaßstäbe, die sich an sozio-kultureller Homogenität, an sozialen Konventionen und Autoritäten sowie an einem traditionellen Berufs- und Sozialethos orientieren.

Es handelt sich bei ihnen also in erster Linie um Bevölkerungssegmente, die objektiv durch die Modernisierungsprozesse der vergangenen Jahrzehnte ökonomisch und sozial ins Hintertreffen geraten sind, sich also mit Statusverlustängsten konfrontiert sehen. Sie haben durch die Ökonomisierung und Deregulierung großer Teile der Sozialwelt im Zuge der neoliberalen Revolution und der damit einhergehenden Internationalisierung vieler akademischer Qualifizierungs- und Berufswege einen schmerzlichen Statusverlust erfahren. Daraus könne, wie Klaus Kraemer (2018) argumentiert, eine „Sehnsucht nach dem nationalen Container“ erwachsen, oder anders ausgedrückt: ein kollektives Bedürfnis nach Rückeroberung oder Wiederaneignung der in Kategorien ethnischer Zugehörigkeit definierten Gesellschaft durch einen in seiner Souveränität erstarkten Nationalstaat.

Doch ungeachtet dieser skizzierten neueren Differenzierung der sozialmoralischen Milieus innerhalb der deutschen Mittelschichten steht dennoch wohl außer Frage, dass es immer noch primär Segmente dieser Klasse sind, die die wichtigsten sozialen Trägergruppen der gegenwärtigen Renaissance des Nationalismus bilden. Die am Faschismus und Nationalsozialismus gewonnene soziologische These, dass der Nationalismus sozialstrukturell primär ein mittelschichtenspezifisches Phänomen sei, ist also keineswegs pauschal zu verwerfen oder etwa obsolet. Neu aber ist, dass sich in jüngerer Zeit innerhalb der Mittelschichten progressive Gegenmilieus herausgebildet haben, die einen bedeutenden Einfluss auf die öffentliche Debatte nehmen. Diese neue sozialmoralische Konstellation lässt eine Wiederkehr faschistoider Politikmuster auf gesamtgesellschaftlicher Ebene eher nicht erwarten, was allerdings nationalistische Tendenzen, die bis weit in die gesellschaftliche Mitte hineinreichen, Deklassierte aller Schichten umfassen und zu einer folgenreichen „Verschiebung der parteipolitischen Machtbalance infolge des Aufstiegs der AfD“ führen (Habermas 2020, S. 41), keineswegs ausschließt.

## Die Corona-Krise als Gefahr für die liberale Demokratie

Mit Blick auf die aktuelle Corona-Krise, die nicht nur Politik, Wirtschaft und Kultur, sondern die Grundstrukturen des menschlichen Zusammenlebens schlagartig und mit heute noch kaum übersehbaren Konsequenzen voraussichtlich umwälzen wird, lässt die Frage nach der Anfälligkeit unserer Gegenwartsgesellschaft für faschistoide Tendenzen noch einmal in einem neuen Licht erscheinen. Mit der Pandemie stellen sich gänzlich neue Probleme, auch und gerade im Hinblick auf die im vorliegenden Beitrag behandelten Sachverhalte. Wurde der beschriebene Trend zur nationalistischen Radikalisierung von Teilen der Mittelschicht durch die Corona-Pandemie gestoppt? Oder droht in Anbetracht des durch die Anti-Corona-Maßnahmen in seiner materiellen Existenz bedrohten Mittelstandes und einem womöglich bevorstehenden Anstieg der Arbeitslosenzahlen eine Eskalation von nationalistischen Konflikten und eine fundamentale Rechtswende der Politik (vgl. Bach 2020; Poppa 2020)?

Die Lage ist unübersichtlich, seriöse Prognosen sind also schwierig. Abschließend sei deshalb nur ein Aspekt aufgegriffen: den für viele überraschenden Einbruch an Zustimmungswerten, den die rechtspopulistischen Parteien neuerdings inmitten der Corona-Krise erleben. Die AfD verlor in den ersten, mitten in der Pandemie stattfindenden Landtagswahlen 2021 erheblich an Stimmenanteilen: in Baden-Württemberg mehr als 5 % und in Rheinland-Pfalz mehr als 4 % (Statista 2021b, c). Wie lässt sich diese überraschende Entwicklung vor dem Hintergrund der obigen Argumentation erklären?

Besinnt sich jetzt etwa die gesellschaftliche Mitte, die in ihren konservativen und von sozialer Deklassierung bedrohten Segmenten strukturell zu einem ethnisch getönten Nationalismus neigt, plötzlich wieder auf politische Mäßigung und auf die Werte der repräsentativen Demokratie? Davon ist nicht auszugehen. Die augenfällige Abkehr der Mittelschichten vom Populismus lässt sich nach meinem Dafürhalten in erster Linie als unmittelbare Folge der Pandemie erklären, denn mit ihrer Verschärfung haben sich die gesellschaftlichen und politischen Machtbalancen gegenüber den Hochzeiten des Rechtspopulismus nochmals tiefgreifend verschoben.

In erster Linie ist dabei an den sich bei der Krisenbewältigung autoritär gebenden Staat und an das entsprechend fürsorglich-paternalistische Auftreten führender Politiker und Politikerinnen, wie dem Bayerischen Ministerpräsidenten Markus Söder, der Bundeskanzlerin Angela Merkel sowie dem Bundesgesundheitsminister Jens Spahn zu denken. Diese präsentieren sich als berufene Problemlöser und Krisenmanager, die allerdings auch nicht vor rigoroser Strenge und Unerbittlichkeit zurückschrecken und Lockdowns, Maskenpflicht, Ausgeh- und Reiseverbote sowie soziale Kontaktbeschränkungen verordnen, was wiederum die alltägliche Interaktions- und Berührungsordnung verändert (Lindemann 2020). Die einschlägigen Entscheidungen, die die Regierungen meist auf dem Verordnungswege treffen, und die mit beispiellosen Einschränkungen der Grundrechte und der Demokratie einhergehen, werden dabei als „alternativlos“ bezeichnet. Hier kündigt sich ein neuartiger Staatsautoritarismus an, der gerade – so meine These – der Mentalität der rechtskonservativen Kräfte in Deutschland sehr weit entgegenkommt. Eine klare Präferenz für eine Diktatur und für einen möglichst „starken Mann“ an ihrer Spitze gehört bekanntlich seit jeher zu den Grundvorstellungen der Rechten (vgl. Zick et al. 2019, S. 121). Diese Haltung wird nun nicht mehr überwiegend nur am rechten Rand des politischen Meinungsspektrums, prominent etwa durch die AfD, artikuliert. Sie droht vielmehr, sich im Rahmen der Pandemiebekämpfung und des Überbietungswettbewerbs eines „illiberalen Verbotspopulismus“ (Merkel 2020) zum dominierenden Narrativ des politischen Zentrums zu entwickeln. Die Politik werde jetzt wieder von „Machern“ geführt, die sich durch persönliche Entschlossenheit und Führungskraft auszeichneten, so die verbreitete Wahrnehmung in der Bevölkerung, wie die oben bereits erwähnten hohen Zustimmungswerte für die Anti-Corona-Maßnahmen insgesamt und für den Bayerischen Ministerpräsidenten zeigen.

Darin könnte sich vielleicht sogar eine überraschende Tendenz zur Manifestation eines weiteren neuen Typs von charismatischen Führern abzeichnen, neben den oben beschriebenen kritischen Internet-Aktivisten, – zu Politikern nämlich, deren Legitimation primär darauf basiert, vermeintlich die Gesundheit und den Zusammenhalt der ganzen Gesellschaft gegen die unsichtbare, aber allgegenwärtige Bedrohung durch das Virus zu verteidigen. Ihr Charisma beruht ebenfalls auf zugeschriebenen außeralltäglichen Qualitäten, die in diesem Fall in einer überlegenen Expertise, einer außergewöhnlichen Entschlusskraft und einer besonderen ethisch-moralischen Rationalität zum Ausdruck kommen. Man könnte diesen Typ als Charismatiker mit gesellschaftstherapeutischer Mission beschreiben. Diesem geht es nicht primär um gängige politische Ziele, wie Wahlsiege oder soziale Gerechtigkeit, sondern um den Erhalt der Volksgesundheit. Dabei rechtfertigt das dominierende Leitkriterium „Es geht um Leben und Tod“ eine massive Einschränkung der Grundrechte, die Etablierung einer die Exekutive ermächtigenden „Parallelrechtsordnung“ (Lepsius 2020), die im Wesentlichen auf Verordnungen basiert, sowie eine Reihe von beispiellosen Kontrolldispositiven, die das öffentliche und private Leben der Bevölkerung stark reglementieren. Das Zurücktreten von parlamentarischen Meinungsbildungs- und Entscheidungsprozessen zugunsten von Ad-hoc-Beschlüssen der Spitzen der Exekutive wird durch die massenmedial angeheizte Wahrnehmung eines extrem hohen Gefährdungs- und Angstniveaus legitimiert. Die Anerkennung des Machtanspruchs der neuen charismatisch-therapeutischen Führer durch die Bürger entspräche dann jener aus „Not und Hoffnung geborene[n] gläubige[n], ganz persönliche[n] Hingabe“, in der Weber bekanntlich das zentrale Strukturmoment der charismatischen Herrschaft sah (Weber 1976, S. 140). Wie bei jeder Form charismatischer Herrschaft entscheidet auch bei der therapeutischen Herrschaft letztlich die persönliche Bewährung der neuen Führer, d.h. ob und inwieweit sich ihre Politik als erfolgreich nach Maßgabe der erklärten Ziele oder der tatsächlichen Aufgaben erweist. Wie weit der charismatische Legitimationsglauben in der gegenwärtigen Lage trägt, wird sich freilich erst in der Zukunft erweisen. Zu konstatieren ist jedoch schon jetzt, dass sich in der Pandemiekrise mit der staatsautoritären Wende vielleicht doch eine neuartige latent charismatische Situation ergeben könnte, die einer außergewöhnlichen Machtfülle einzelner Politiker den Weg ebnen würde. Während sich in der rechtspopulistischen Szene in Deutschland bisher keine genuin charismatischen Führer mit gesamtgesellschaftlicher Strahlkraft profilieren konnten, treten solche neuerdings auf der offiziellen Staatsbühne auf – damit eröffnen sich unerwartete Chancen für die Verwirklichung eines politischen Ideals der Rechten. Das Paradox, oder dialektisch gewendet, die „List der Geschichte“ besteht jedoch darin, dass die Pandemiekrise und die mit ihr einhergehende staatsautoritäre Wende nicht den rechten Parteien zugutekommt, sondern dem politischen Zentrum.

## Schlussbetrachtung

Die Abgrenzung vom historischen Faschismus und Nationalsozialismus gehört zum Selbstverständnis Nachkriegsdeutschlands, weshalb sich dessen rechtsstaatliche Demokratie immer auch als Gegenentwurf zu den moralischen Pervertierungen der Diktaturen zu bewähren hat. Das öffnet einem Antifaschismus im öffentlichen Diskurs Tür und Tor, der sich häufig genug in purer Rhetorik erschöpft. Namentlich in gesamtgesellschaftlichen Krisensituationen, wenn mehr oder weniger diffuse Bürgerproteste, wie die PEGIDA-Bewegung, öffentlich nationalistische, fremdenfeindliche oder auch antisemitische Töne anschlagen, sich rechtspopulistische Parteien, wie die AfD, bilden oder sich eine neuartige, politisch nicht eindeutig zuordenbare außerparlamentarische Systemgegnerschaft öffentlich Gehör zu verschaffen versucht, wie die Querdenker-Bewegung, ist ein genereller Faschismusverdacht schnell zur Hand. Der Faschismusvorwurf konnte sich auf diese Weise als nahezu beliebig aufgreifbares Stereotyp in der politischen und massenmedialen Auseinandersetzung in Deutschland etablieren.

Wie sich die vermeintliche Gefährdung von Demokratie und Rechtsstaatlichkeit gegenwärtig in Deutschland darstellt und wie sie in historisch vergleichender Perspektive zu beurteilen ist, erfordert jedoch eine differenziertere Betrachtung, die sich auf einem soliden historisch-soziologischen Terrain zu bewegen hat. Einer der wichtigsten soziologischen Referenzautoren für ein derartiges Vorhaben ist zweifellos Max Weber, und zwar aus methodologischen wie herrschaftssoziologischen Gründen. Ein gemäß seiner idealtypischen Methode konzipiertes Modell des Faschismus, das sogenannte „faschistische Minimum“, ist hervorragend als *tertium comparationis* für einen methodisch-systematischen Vergleich von politischen Herrschaftsstrukturen in historischer Perspektive geeignet. Insbesondere Webers Grundbegriffe der Herrschaftssoziologie, wie der Parteienbegriff, das Modell der charismatischen Herrschaft und das Paradigma der Bürokratie, können dabei ihre besondere analytische Fruchtbarkeit erneut unter Beweis stellen.

Betrachtet man die soziopolitischen Gegenwartstendenzen, die sich mit dem nationalistischen Populismus verbinden, unter diesem Blickwinkel, so gelangt man zu dem Befund, dass, zugespitzt formuliert, von einer faschistischen oder faschistoiden Tendenz im heutigen Deutschland objektiv schwerlich die Rede sein kann. Wesentliche Struktureigenschaften, die für den historischen Faschismus fundmental waren, wie etwa der landesweite Einsatz paramilitärisch organisierter Gewalt oder der Aufstieg charismatischer Führer in Verbindung mit politisch motivierter Gewalt auf der Straße lassen sich für die heutige populistische Bewegung hierzulande bisher nicht erkennen. Und auch die nationalistischen Radikalisierungspotenziale innerhalb des deutschen Mittelstandes, der die sozialstrukturelle Manövriermasse für den Aufstieg des Nationalsozialismus bildete, bestehen gegenwärtig nur bei bestimmten, konservativen Segmenten dieser sozialen Schicht, während andere Segmente zu dezidiert liberalen und kosmopolitischen Politikentwürfen neigen, was zu einer beachtlichen inneren Heterogenität und Widersprüchlichkeit in den Wertorientierungen dieser Schicht geführt hat.

Dessen ungeachtet soll hier keineswegs einer naiven Sorglosigkeit das Wort geredet werden. Tendenzen zur Radikalisierung von nationalistischen und antiliberalen Gruppierungen sind im heutigen Deutschland nicht von der Hand zu weisen. Deren Dynamik ist aber definitiv nicht mit der Sprengkraft der entsprechenden Bewegungen in der Weimarer Republik vergleichbar. Das ist bisher wohl in erheblichem Maße der Resilienz der politischen Institutionen, insbesondere des Rechtsstaates und der Demokratie zuzuschreiben. Allerdings ist in Anbetracht der aktuellen Corona-Krise gerade bei den politischen Institutionen und beim politischen Klima in der Bundesrepublik insgesamt eine präzedenzlose staatsautoritäre und illiberale Trendumkehr zu beobachten, die als durchaus besorgniserregend eingestuft werden muss. Es wird zu prüfen sein, inwieweit die damit einhergehenden fundmentalen gesellschaftlichen und politisch-institutionellen Umbrüche, die in einer bisher unbekannten Weise die unmittelbare Interaktionsebene menschlichen Verhaltens nach Maßgabe hygienepolitischer Vorgaben (von „social distancing“ bis hin zur Aussetzung von Grundrechten) zu regulieren trachten, mit den herrschaftssoziologischen Kategorien Max Webers analysiert werden können. Es ist allerdings zu erwarten, dass mit den aktuellen Tendenzen, die auf einen „Notstands-Staat“ (Gumbrecht 2020) hinauslaufen und in einem neuartigen, im Medium von Angst und Fürsorge operierenden Politikstil münden könnten, gerade Webers Herrschaftssoziologie wieder neue Aktualität gewinnt.

## Footnotes

1 Die nachfolgenden Überlegungen konzentrieren sich primär auf Deutschland. Fallweise herangezogene internationale Vergleiche – sei es mit dem historischen Faschismus Italiens, sei es mit anderen europäischen Ländern – dienen lediglich der Illustration der Ausführungen.

2 Seit Ausbruch der Corona-Krise im Frühjahr 2020 sind die Zustimmungswerte bei Meinungsumfragen für rechtspopulistische Parteien in Deutschland sogar tendenziell rückläufig. Wenn derzeit eine Transformation der Demokratie in Richtung eines ausgeprägten Staatsautoritarismus zu beobachten ist, dann ist diese Entwicklung auf die Errichtung einer neuartigen „Parallelrechtsordnung“ (Lepsius 2020) zurückzuführen, die unter infektionsschutzpolitischen Aspekten zentrale Grundrechte außer Kraft setzt und durch Kontaktverhinderungsregulierungen und Massenerziehungsmaßnahmen (Hygienevorschriften wie Masken- und Distanzierungspflicht) das alltägliche Verhalten der Menschen lenkt und dadurch substanziell verändert. Dabei handelt es sich um folgenreiche Eingriffe in die *conditio humana*, in die Grundvoraussetzungen des Menschseins (Burchardt 2020), deren Konsequenzen für das gesellschaftliche Zusammenleben, die Politik und die alltäglichen Verhaltenskanons derzeit noch gar nicht abzusehen sind.

3 Anders stellt sich die Situation in anderen Ländern Europas dar, in denen rechtspopulistische Parteien bereits an der Regierung sind bzw. autoritäre Regime herrschen, wie etwa in Ungarn, Polen oder in der Türkei.

4 Das könnte sich freilich im weiteren Verlauf der COVID-19-Pandemie ändern, weil gesamtgesellschaftliche Krisen stets einen fruchtbaren Boden für charismatische Tendenzen darstellen. Und eine vergleichbare Krise, wie die Corona-Krise, hat die Welt seit dem Ende des Zweiten Weltkrieges nicht erlebt. Hinweise darauf finden sich in der Bereitschaft von Gegnern der Corona-Maßnahmen (den „Querdenkern“), neuen, in alternativen (Internet‑)Foren mobilisierenden Anführern, die oft Verschwörungstheorien anhängen und eine (Selbst‑)Heroisierung praktizieren, gläubig zu folgen. Darin manifestiert sich auch eine für charismatische Bewegungen typische Entfremdung gegenüber den politischen Institutionen des politischen Establishments, den tradierten Formen der Repräsentation, den Volksparteien sowie den Leitmedien (vgl. Schließler et al. 2020, S. 304).

5 Die Sitzungen des „Corona-Ausschusses“ unter der Leitung des Rechtsanwaltes Reiner Füllmich beispielsweise dauern jeweils mehrere Stunden. Auch die über interaktive Live-Streams hergestellte Internet-Präsenz vieler Anti-Corona-Aktivisten kennt keine festen Zeitformate, sondern wird oft in deren Alltagsleben integriert bzw. mit öffentlichen Aktionen, wie Demonstrationen, synchronisiert. In den nahezu täglich produzierten populären Inernet-Videos des österreichischen Psychiaters und Maßnahmengegners Raphael Bonelli etwa verschwimmen die Grenzen zwischen Privatleben und politischer Öffentlichkeit dadurch immer wieder, dass seine Auftritte u.a. in seinen Wohnräumen aufgenommen und dabei gelegentlich auch seine Ehefrau und Kinder mit einbezogen werden. Dadurch entsteht der Eindruck einer familiär-vertraulichen Leidens- und Widerstandgemeinschaft, als Gegenentwurf zu der als „kalt“ und „unbarmherzig“ empfundenen Politik der herrschenden Institutionen.

6 Der Umfrage von Nachtwey et al. (2020, S. 51) in der Schweiz und Deutschland zufolge handelt es sich bei der Querdenker-Bewegung hauptsächlich um eine von relativ älteren Akademikern geprägte Bewegung: Das Durchschnittsalter beträgt 47 Jahre, 31 Prozent geben das Abitur, 34 Prozent einen Studienabschluss als höchsten Bildungsabschluss an. Der Anteil der Selbstständigen ist mit 25 Prozent deutlich höher als in der Gesamtbevölkerung.
